# Is Canine Prostate-Specific Esterase a Reliable Marker for Benign Prostatic Hyperplasia Progression in Dogs?

**DOI:** 10.3390/ani15111614

**Published:** 2025-05-30

**Authors:** Florin-Petrișor Posastiuc, Nicolae-Tiberiu Constantin, Guillaume Domain, Lotte Spanoghe, Ann Van Soom, Alexandru Ilie Diaconescu, Mario-Darius Codreanu

**Affiliations:** 1 Department of Internal Medicine, Reproduction and Population Medicine, Faculty of Veterinary Medicine, Ghent University, Salisburylaan 133, 9820 Merelbeke, Belgium; florin.posastiuc@ugent.be (F.-P.P.); guillaume.domain@ugent.be (G.D.); lotte.spanoghe@ugent.be (L.S.); ann.vansoom@ugent.be (A.V.S.); 2 Department of Clinical Sciences II, Faculty of Veterinary Medicine, University of Agronomic Sciences and Veterinary Medicine of Bucharest, 105 Blvd. Splaiul Independentei, 050097 Bucharest, Romania; alexandru.diaconescu@fmvb.usamv.ro (A.I.D.); mario.codreanu@fmvb.usamv.ro (M.-D.C.)

**Keywords:** BPH, CPSE, disease progression, biomarker, clinical severity, ultrasonography

## Abstract

Benign prostatic hyperplasia (BPH) is a common condition in intact male dogs, often progressing from subclinical to symptomatic stages with increasing clinical and structural impact. This study evaluated canine prostate-specific esterase (CPSE) as a biomarker for BPH progression, focusing on clinical severity, complexity, and ultrasonographic features. Seventy-one dogs were included: subclinical-BPH (n = 14), clinical-BPH (n = 26), BPH-prostatitis (n = 9), and controls (n = 22). CPSE levels, measured using a canine-specific enzyme-linked immunosorbent assay, were significantly correlated with clinical severity (*ρ* = 0.800, *p* ≤ 0.001) and complexity (*ρ* = 0.818, *p* ≤ 0.001). CPSE predicted mild (OR = 1.260, B = 0.231, *p* ≤ 0.001) and moderate severity (OR = 1.300, B = 0.262, *p* ≤ 0.001), as well as low (OR = 1.225, B = 0.203, *p* ≤ 0.05), moderate (OR = 1.235, B = 0.211, *p* ≤ 0.01), and high (OR = 1.346, B = 0.297, *p* ≤ 0.001) clinical complexity. CPSE showed a trend toward predicting structural alterations (OR = 1.227, B = 0.204, *p* = 0.069) and was associated with larger stippled areas, increased prostatic volume, and larger cysts/abscesses (all *p* ≤ 0.001). CPSE appears to be a promising marker for BPH progression.

## Simple Summary

Benign prostatic hyperplasia (BPH) is a common condition in non-castrated male dogs, characterized by prostate enlargement. This condition can range from having no symptoms to more severe forms. This study aimed to test if a blood marker, called canine prostate-specific esterase (CPSE), can help monitor how BPH progresses in dogs. The researchers studied 71 dogs, grouped based on the severity of the condition, from simple to more complicated forms. They found that higher levels of this marker were linked to more severe symptoms and greater changes in the prostate, which could be seen through ultrasound. Additionally, CPSE was able to predict the extent of these changes at different stages of the disease. This research suggests that measuring CPSE can be helpful to monitor the evolution of BPH in dogs, providing a valuable tool for better monitoring and treatment of the condition.

## 1. Introduction

Benign prostatic hyperplasia (BPH) is the most common reproductive tract disorder in intact male dogs, with nearly 90% of individuals affected by eight years of age [1]. BPH typically begins as a subclinical condition, eventually evolving into a symptomatic form characterized by a wide range of clinical manifestations and complications, which can significantly impair the patient’s quality of life [2,3,4]. As the disease progresses, the prostatic structure may undergo various alterations, with cyst formation being one of the most common findings [5]. The dynamic evolution of BPH is further complicated by the predisposition to prostatitis, a condition that, in some instances, may be life-threatening [6,7]. In fact, the concomitant evolution of BPH and prostatitis is common and can lead to more severe symptoms while accelerating structural damage within the prostate, such as the septic transformation of cysts into abscesses that may rapidly enlarge and necessitate a different therapeutic approach [8,9].

Considering the progressive nature of BPH, predictive markers for clinical or structural implications become highly valuable for the overall management of this disorder. The canine prostate-specific esterase (CPSE) is the major secretory product of the canine prostate, sharing several similarities with prostate-specific antigen (PSA) in men, including very similar hormonal regulation [4,10]. While PSA has been extensively studied as a progression factor for BPH in men [11,12,13], the role of CPSE in dogs has primarily been explored in the context of diagnosis rather than as a predictor of disease evolution [14,15,16,17].

The aim of this study was to evaluate CPSE as a predictive marker for BPH progression in intact male dogs across different stages, including the concomitant evolution of BPH and prostatitis. Specifically, the study sought to determine whether CPSE levels can forecast the degree of clinical severity, symptom complexity, and ultrasonographic structural alterations, ultimately providing a dynamic tool for early detection, monitoring disease evolution, and guiding tailored therapeutic interventions.

## 2. Materials and Methods

This study was designed as a cross-sectional multicenter observational study, collecting data from intact male dogs evaluated at a single time point. Subjects were referred to either the Small Animal Reproduction Department at the Faculty of Veterinary Medicine, Ghent University (Belgium), or the University of Agronomical Sciences and Veterinary Medicine of Bucharest (Romania) for the investigation of a suspected prostatic disorder or for a comprehensive breeding soundness evaluation.

### 2.1. Patient Evaluation, Inclusion and Grouping

#### 2.1.1. History and Clinical Examination

All subjects underwent a thorough anamnesis and clinical examination. Records were taken on the twenty-three prostate-related clinical symptoms (Table S1) as defined by Cazzuli et al. (2023) [18] with the addition of hematospermia. Semen collection and analysis were performed exclusively in asymptomatic patients with no clinical signs or history suggestive of prostatic disease, in order to exclude hematospermia. Dogs with no blood in their ejaculate were classified as asymptomatic. In contrast, dogs exhibiting at least one symptom were classified as symptomatic.

#### 2.1.2. Ultrasound Evaluation

All patients underwent an ultrasound examination of the prostate and testicles using either a MyLab™50 XVision (Esaote, Genova, Italy) or MyLab Six system (Esaote, Genova, Italy). In all cases, a microconvex curved array transducer with a frequency range of 5.0–9.0 MHz was used. The examinations were performed by two trained veterinarians following a predefined protocol to ensure standardized assessment [19].

The prostate was measured in two planes: longitudinal and transverse. The length (L), width (W), and depth (DL depth on the longitudinal scan; DT depth on the transverse scan) were recorded, and the actual volume of the prostate gland was then calculated using the formula proposed by Atalan et al. (1999) [20]:

Actual volume (cm^3^) = 0.487 × L × W × (DL + DT):2 + 6.38 (1)

Additionally, the expected prostatic volume was determined based on the dog’s age (A) and body weight (BW), using the formula proposed by Ruel et al. (1998) [21]:

Expected volume (cm^3^) = (0.867 × BW) + (1.885 × A) + 15.88 (2)

Dogs with an actual prostatic volume greater than the expected one were classified as having prostatomegaly.

Moreover, the ultrasonographic structure of the prostate was evaluated. The normal prostate appearance was defined according to previous studies as having a uniform, symmetrical bilobed shape on transversal scans and an ovoid shape on longitudinal scans, with smooth echotexture and an echogenicity comparable to that of the spleen [4,22]. Any intra/paraparenchymal hypoechoic structures compatible with fluid cavitations were noted. The presence of these alterations, either alone or in combination with other focal changes, along with any deviation from the normal echotexture, degree of parenchymal stippling, or homogeneity, as previously defined by Russo et al. (2012) [23], qualified individuals as having an altered prostatic ultrasonographic appearance.

#### 2.1.3. Prostatic Fine Needle Aspiration (FNA) and Cytology

Patients with an altered prostatic ultrasound appearance, as previously defined, regardless of prostate volume, underwent prostatic FNA with subsequent cytological evaluation. The procedure was carried out following the technique described by Kustritz et al. (2006) [24], under ultrasound guidance and without sedation. Moreover, if intra- or paraprostatic fluid accumulations were identified on ultrasound, additional aspiration was performed to determine their nature, distinguishing between an abscess or a cyst. Slides were, thereafter, outsourced and analyzed by trained personnel.

#### 2.1.4. Blood Sampling

All patients underwent blood sampling during the same visit via venipuncture of the cephalic vein. The blood was collected into lithium heparin tubes (BD Vacutainer, Plymouth, UK), and immediately centrifuged at 2500× *g* for 10 min. The resulting plasma was then stored at −20 °C for subsequent CPSE analysis.

#### 2.1.5. Patient Inclusion and Grouping

Only dogs that followed the already mentioned evaluation steps and qualified for one of the four defined groups were finally admitted:

- Subclinical BPH: asymptomatic dogs with prostatomegaly
  ○ If an altered ultrasound appearance was observed, inclusion in this group was confirmed by cytology, requiring the presence of only large clusters of prostatic cells with normal characteristics as defined by Laurusevičius et al. (2024) [16].
- Clinical BPH: symptomatic dogs with prostatomegaly
  ○ If an altered ultrasound appearance was observed, inclusion was confirmed by cytology, using the same criteria as for subclinical BPH.
- BPH-Prostatitis: dogs with prostatomegaly and cytological evidence of prostatic inflammation and/or abscessation:
  ○ Cytology suggestive of both BPH and prostatitis, characterized by high numbers of neutrophils and/or macrophages in addition to large epithelial cell clusters, with or without free or intracellular bacteria [15].
  ○ Presence of degenerated neutrophils, with or without bacteria, in the aspirated fluid, indicating prostatic abscessation [25].
- Control: Asymptomatic dogs with no prostatomegaly and a normal prostatic ultrasound appearance.

### 2.2. Disease Progression Evaluation

#### 2.2.1. Clinical Grading

Clinical progression was assessed based on two aspects: clinical complexity and severity. Clinical complexity was defined based on the diversity of symptom categories identified in each case, following the classification described by Cazzuli et al. (2023) [18] and detailed in Table S1. Specifically, cases with symptoms from three different categories were classified as having high clinical complexity, those with symptoms from two categories as moderate complexity, and those with symptoms from one category as low complexity. Cases without any symptoms (asymptomatic BPH) were designated as having no complexity. Disease severity was evaluated using the index proposed by Zambelli et al. (2012) [26] and later adapted by Ruetten et al. (2021) [8]. This adaptation resulted in the following final severity categories: asymptomatic (score 11), mild (12–22), moderate (23–33), and severe (34–44), which were also applied to our study. For clarity and consistency, this index will, hereafter, be referred to as the Zambelli Index Score (ZIS).

#### 2.2.2. Degree of Structural Alteration

The degree of prostate enlargement was assessed based on the ratio between the actual prostate volume and the expected prostatic volume; hereafter referred to as the actual:expected volume ratio.

The ultrasound appearance was evaluated according to the criteria previously detailed by Russo et al. (2012) [23] and applied by Niżański et al. (2020) [19]. Thus, each prostate was evaluated qualitatively based on four criteria: background echotexture (normal, hyperechoic or hypoechoic), parenchymal stippling (regular, increased, or coarse), general appearance (homogeneous or heterogeneous), and focal changes (cysts, mineralized opacities, or focal hypoechoic lesions). The degree of structural alteration was then classified based on the number of abnormal features observed, resulting in four categories: no structural alteration (no abnormal features), mild structural alteration (1–2 abnormal features), moderate structural alteration (3 abnormal features), and severe structural alteration (>3 abnormal features).

**Figure 1:**
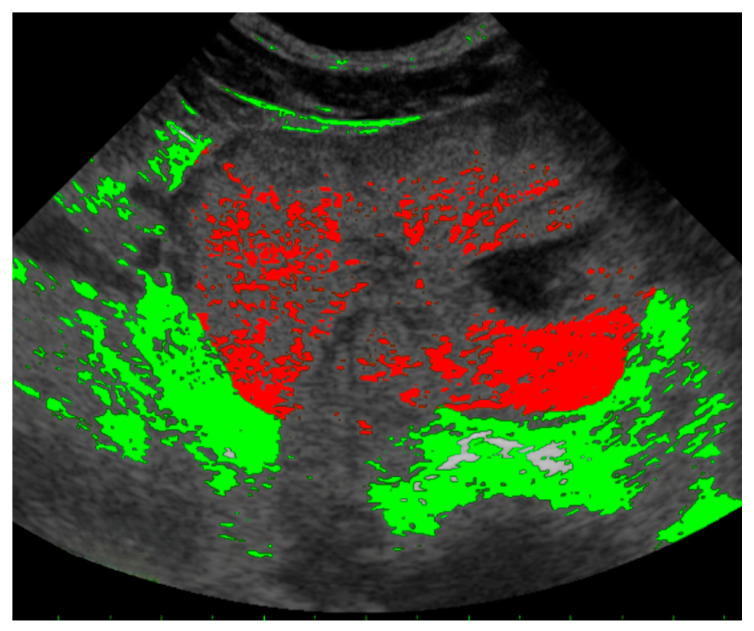
Ultrasound image of the prostatic parenchyma with pixel analysis performed within a defined region of interest (ROI). Stippled areas were identified based on a standardized pixel intensity threshold. Red indicates stippled areas within the ROI, while green marks regions excluded due to their location outside the ROI.

The degree of parenchymal stippling was quantified using pixel-based image analysis through the Leica Application Suite (LAS, version 4.13, Wetzlar, Germany). Regions of interest (ROIs) were defined by manually outlining the prostatic silhouette on both transverse and longitudinal ultrasound sections. The total ROI area was calculated and expressed in pixel². Areas of increased echogenicity within the prostate parenchyma, operationally defined as stippled regions, were detected using a standardized pixel intensity threshold, with color correction via white balancing, as described in previous protocols utilizing the same software [27,28] (Figure 1). The stippled area was quantified and expressed as a percentage of the total ROI area for both transverse and longitudinal images. For each individual, the average percentage from the two sections was used for analysis.

Cysts or abscess size was measured at its maximum diameter for each individual and categorized into three classes: ≤1 cm (small), 1–2 cm (medium), and >2 cm (large). If present, paraprostatic cysts or abscesses were also recorded.

All ultrasound evaluations were performed by two trained operators. However, the qualitative structural characterization of the images and the pixel-based quantification were conducted by a single operator who was blinded to the group allocation of the dogs.

### 2.3. CPSE Levels Evaluation

CPSE levels were quantified in stored plasma samples using a canine-specific sandwich-type immunoassay kit (CPSE ELISA Kit EK762313, AFG Bioscience LLC., Northbrook, IL, USA), following the manufacturer’s instructions. Briefly, after serial dilution, 50 μL of each standard was added to the 96-well plate in duplicate. Using a multichannel pipette, 40 μL of diluent was added to the sample wells, followed by 10 μL of each plasma sample. After incubation at 37 °C for 40 min, the plate was washed, and 50 μL of horseradish peroxidase-conjugated antibody was added to each well (excluding the blank). Following another incubation and wash step, 50 μL of Chromogen A and 50 μL of Chromogen B were added, and the plate was incubated in the dark for 20 min. Finally, 50 μL of stop solution was added, and absorbance was measured at 450 nm using a Multiskan™ GO spectrophotometer (Thermo Fisher Scientific, Vantaa, Finland). The detection limit of the kit was 0.31 ng/mL, while the intra- and inter-assay coefficients of variation were ≤8% and ≤10%, respectively.

### 2.4. Statistical Analysis

The data were analyzed using IBM SPSS version 26.0 for Windows (IBM Corp., Armonk, NY, USA). The normality of the variables was evaluated with the Shapiro–Wilk test, applying a significance level of α = 0.05. Data are expressed as median and interquartile range (IQR), with the 25th and 75th percentiles reported. Outliers within each group were identified using the IQR method. When the data were found to be normally distributed within two independent groups, distribution was evaluated using an independent samples *t*-test. The independent samples Mann–Whitney *U* and Kruskal–Wallis tests were applied to determine differences in the distribution of skewed data, followed by pairwise comparisons with significance values adjusted using the Bonferroni correction. The Chi-Square Test was applied to assess associations between categorical variables. When the conditions for this test were not met, the Fisher–Freeman–Halton Exact test, along with Monte Carlo simulations, were used as alternatives. Spearman’s rank correlation was used to examine the associations between the different numerical variables. Multinomial logistic regression models were applied to assess the predictive value of CPSE levels on clinical severity/complexity or ultrasonographical structural alteration. Potential confounders considered for these models were age, weight, and breed. The inclusion of the confounders in the models was based on their correlation with CPSE, their variability within the population, and their potential to improve the model’s explanatory power.

## 3. Results

### 3.1. Study Population

Seventy-nine dogs followed the entire protocol as defined by this study’s experimental design. However, eight asymptomatic dogs with an altered prostatic ultrasonographic appearance could not be assigned to any of the predefined groups. In all these cases, secondary to FNA-cytologies prostatic cells exhibited characteristics of squamous metaplasia, as previously described by Pinheiro et al. (2017) [15], including large, clear to basophilic cytoplasm and a small, dense nucleus, with no evidence of BPH. Five of the previously mentioned eight dogs presented with a mass in one of the testicles. In three subjects, a Sertoli cell tumor was diagnosed, while the remaining two cases were identified as mixed-type tumors containing a Sertoli cell tumor component. Thus, seventy-one dogs from fourty-one different breeds and a median age of 5.83 years (IQR: 3.25–9.33) were finally included as follows: subclinical BPH (n = 14), clinical BPH (n = 26), BPH-Prostatitis (n = 9), control (n = 22). The detailed age, weight, and breed distribution are presented in Table S2.

### 3.2. CPSE Levels

**Figure 2:**
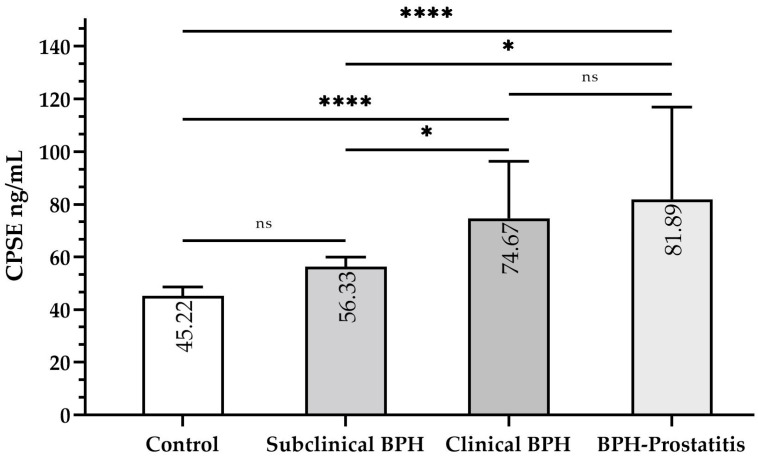
Bar graph displaying median CPSE levels (ng/mL) and interquartile ranges (IQR), along with intergroup differences. Statistical significance is indicated above the bars (* *p* < 0.05, **** *p* < 0.0001, ns = not significant); CPSE = canine prostatic specific esterase; BPH = benign prostatic hyperplasia.

Median CPSE levels varied significantly across specific groups (Figure 2). Levels in control dogs (45.22 ng/mL, IQR: 43.00–48.56) were significantly lower than in clinical BPH (74.67 ng/mL, IQR: 62.44–96.33, *p* ≤ 0.0001) and BPH-Prostatitis (81.89 ng/mL, IQR: 65.78–116.89, *p* ≤ 0.0001). However, they did not significantly differ from subclinical BPH dogs (56.33 ng/mL, IQR: 48.56–59.94, *p* > 0.05). CPSE levels in subclinical BPH were significantly lower than in clinical BPH (*p* ≤ 0.05) and BPH-Prostatitis (*p* ≤ 0.05), but no significant difference was observed between the latter two groups (*p* > 0.05).

**Figure 3:**
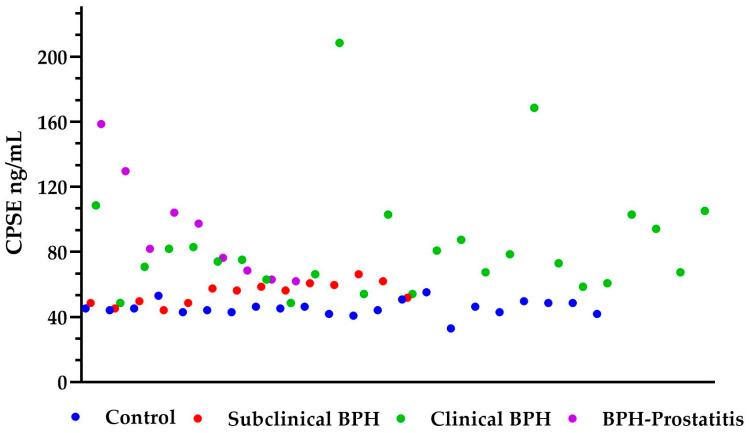
Scatter plot showing the distribution of CPSE levels (ng/mL) across all four groups. Each dot represents an individual measurement. CPSE = canine prostatic specific esterase; BPH = benign prostatic hyperplasia.

Potential outliers were identified: one in the control group (33.00 ng/mL), two in the clinical BPH group (208.56 ng/mL and 168.56 ng/mL), and one in the BPH-Prostatitis group (158.56 ng/mL) (Figure 3). These values were retained in the analysis as they were considered biologically plausible and may reflect real clinical variation within the population. A sensitivity analysis excluding these outliers was performed, confirming that the statistical significance of group differences remained unchanged.

The weight of the participants was not significantly correlated with CPSE levels (*ρ* = 0.015, *p* > 0.05). However, age showed a significant positive correlation with CPSE levels (*ρ* = 0.497, *p* ≤ 0.01), and, thus, its effect was evaluated in the subsequent predictive models. Breed was excluded as a confounder due to its high variability within the studied population, which could increase the risk of overfitting and potentially compromise the model’s stability and generalizability.

### 3.3. Clinical Severity and Complexity

Due to the asymptomatic presentation in both the control and subclinical BPH groups, the ZIS remained constant at 11, resulting in highly significant differences when compared to the clinical BPH (17, IQR: 14–19, *p* ≤ 0.001) and BPH-Prostatitis (20, IQR: 17–25.5, *p* ≤ 0.001) groups. When comparing ZIS between clinical BPH and BPH-Prostatitis, it was found to be significantly lower in the former (*p* ≤ 0.05). Moderate severity was more frequent in BPH-Prostatitis cases (44.4%) compared to clinical BPH (3.8%, *p* ≤ 0.05), while mild severity remained the predominant category in both groups (*p* ≤ 0.05). High severity was not observed in either group. Interestingly, no significant association was found between the degree of clinical complexity and the clinical BPH or BPH-Prostatitis status (*p* > 0.05). In spite of this, dogs with BPH-Prostatitis displayed a significantly higher median number of symptoms (9, IQR: 7.5–13) compared to those with clinical BPH (5.5, IQR: 3.75–6.25, *p* ≤ 0.01).

Within all four groups, CPSE levels were positively correlated with both the number of symptoms (*ρ* = 0.852, *p* ≤ 0.001) and ZIS (*ρ* = 0.825, *p* ≤ 0.001). Moreover, higher CPSE values were characteristic of more complex (*ρ* = 0.818, *p* ≤ 0.001) and more severe (*ρ* = 0.800, *p* ≤ 0.001) clinical presentations.

**Figure 4:**
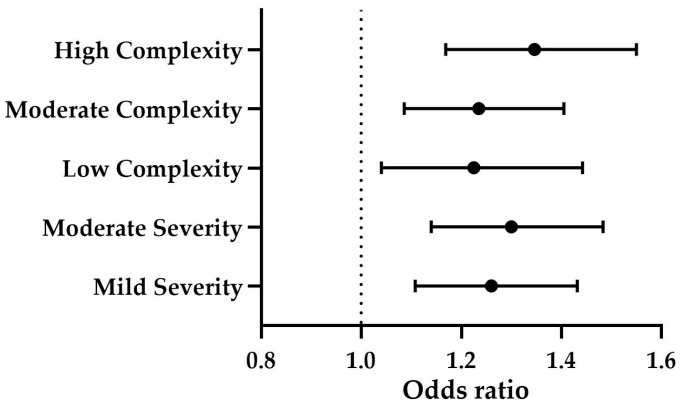
Forest plot showing the odds ratios (ORs) and 95% confidence intervals (CIs) for the predictive value of CPSE levels on clinical severity (mild and moderate severity) and complexity (low, moderate, and high complexity). The solid circles represent the ORs, and the whiskers indicate the CIs. The dotted line at OR = 1 serves as a reference. ORs greater than 1 indicate a positive predictive effect. CPSE = canine prostatic specific esterase.

The levels of CPSE significantly predicted both clinical complexity (*p* ≤ 0.001) and severity (*p* ≤ 0.001). Specifically, CPSE levels were positively associated with low complexity (OR = 1.225, B = 0.203, *p* ≤ 0.05), moderate complexity (OR = 1.235, B = 0.211, *p* ≤ 0.01), and high complexity (OR = 1.346, B = 0.297, *p* ≤ 0.001) (Figure 4). Additionally, CPSE was a significant predictor of mild severity (OR = 1.260, B = 0.231, *p* ≤ 0.001) and moderate severity (OR = 1.300, B = 0.262, *p* ≤ 0.001) (Figure 4). The effect of age was not statistically significant (*p* > 0.05).

### 3.4. Ultrasonographic Structural Alterations

**Figure 5:**
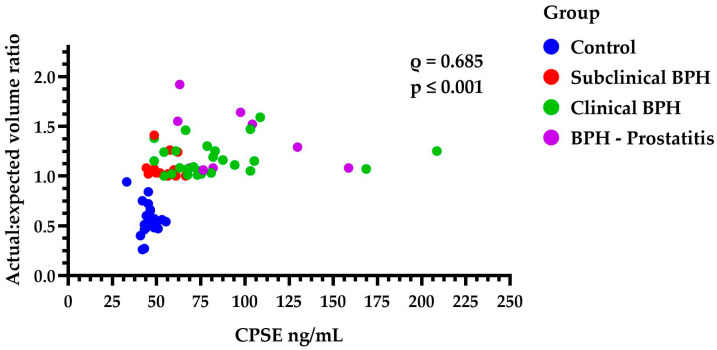
Scatter plot showing the distribution of the actual/expected prostatic volume ratio across all four groups and its positive correlation (ρ = 0.685, *p* ≤ 0.001) with CPSE levels (ng/mL). Each dot represents an individual measurement. CPSE = canine prostatic specific esterase; BPH = benign prostatic hyperplasia.

Prostate enlargement, as measured by the actual/expected volume ratio, was significantly lower in the control group (0.53, IQR: 0.45–0.66) compared to the other three groups: subclinical BPH (1.03, IQR: 1.01–1.12, *p* ≤ 0.001), clinical BPH (1.13, IQR: 1.04–1.25, *p* ≤ 0.001), and BPH-Prostatitis (1.29, IQR: 1.08–1.59, *p* ≤ 0.001). When comparing subclinical BPH, clinical BPH, and BPH-Prostatitis with each other, no statistically significant differences in the degree of prostatic enlargement were found between any of the groups (*p* > 0.05). However, a significant positive correlation was observed between CPSE levels and the actual/expected prostate volume ratio (*ρ* = 0.685, *p* ≤ 0.001), indicating that higher CPSE levels were associated with greater prostate enlargement (Figure 5).

Cyst/abscess presence was significantly associated with patient group category (*p* ≤ 0.001). It was highly specific to clinical BPH (100%) but less specific for the BPH-Prostatitis group (88.9%) and subclinical BPH (71.4%), while no cysts or abscesses were observed in the control group. Moreover, the maximal cyst/abscess size was significantly larger in the clinical BPH group (1.15 cm, IQR: 0.58–1.86) compared to the subclinical BPH (0.18 cm, IQR: 0–0.32, *p* ≤ 0.01), but no significant difference was found between clinical BPH and the BPH-Prostatitis (1.82 cm, IQR: 0.22–3.77, *p* > 0.05). When categorizing sizes, a strong group association was found (*p* ≤ 0.001). According to the latter, subclinical BPH primarily exhibited small structures (71.4%), while clinical BPH and BPH-Prostatitis both exhibited a similar distribution of cyst/abscess sizes, with medium structures found in 30.8% and 33.3%, respectively, and large in 23.1% and 33.3%. Higher CPSE levels were significantly associated with the presence of intraprostatic cysts/abscesses (*p* ≤ 0.001), and larger structures were observed in cases with higher CPSE levels (*ρ*  = 0.803, *p* ≤ 0.001). Seven dogs exhibited an additional paraprostatic cyst/abscess alongside at least one minimal intraprostatic fluid accumulation. However, CPSE levels did not significantly differ between dogs with only intraprostatic cysts/abscesses and those with an additional paraprostatic structure (*p* > 0.05).

**Figure 6:**
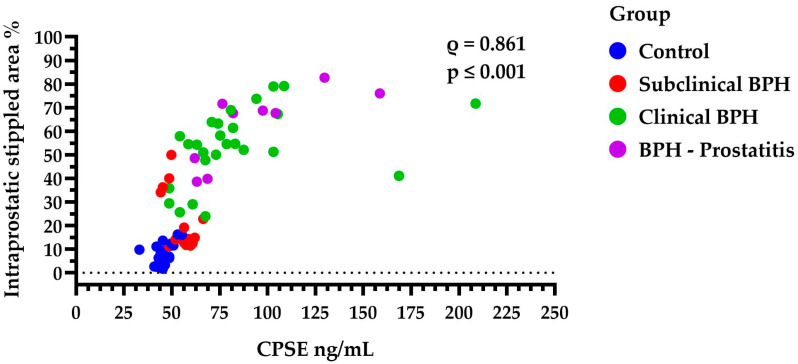
Scatter plot showing the distribution of the intraprostatic stippled area (%) across all four groups and its correlation with CPSE levels (ng/mL). Larger stippled areas were strongly correlated with higher CPSE levels (ρ  = 0.861, *p* ≤ 0.001). Each dot represents an individual measurement. CPSE = canine prostatic specific esterase; BPH = benign prostatic hyperplasia.

The intraprostatic stippled area was significantly higher in the clinical BPH dogs (54.51%, IQR: 46.06–64.72) compared to both the subclinical BPH group (14.6%, IQR: 12.29–34.63, *p* ≤ 0.01) and the Control group (6.65%, IQR: 3.35–11.21, *p* ≤ 0.001). However, no significant difference was found between Clinical BPH and BPH-Prostatitis (67.67%, IQR: 44.19–73.81, *p* > 0.05), nor between the Control and Subclinical BPH groups (*p* > 0.05). Additionally, larger stippled areas were strongly correlated with higher CPSE levels (*ρ*  = 0.861, *p* ≤ 0.001) (Figure 6). This may be even more noteworthy given that the percentage of the stippled area appears to be positively correlated with clinical complexity (ρ = 0.858, *p* ≤ 0.001), clinical severity (ρ = 0.819, *p* ≤ 0.001), and ultimately with the ZIS values (ρ = 0.848, *p* ≤ 0.001).

**Figure 7:**
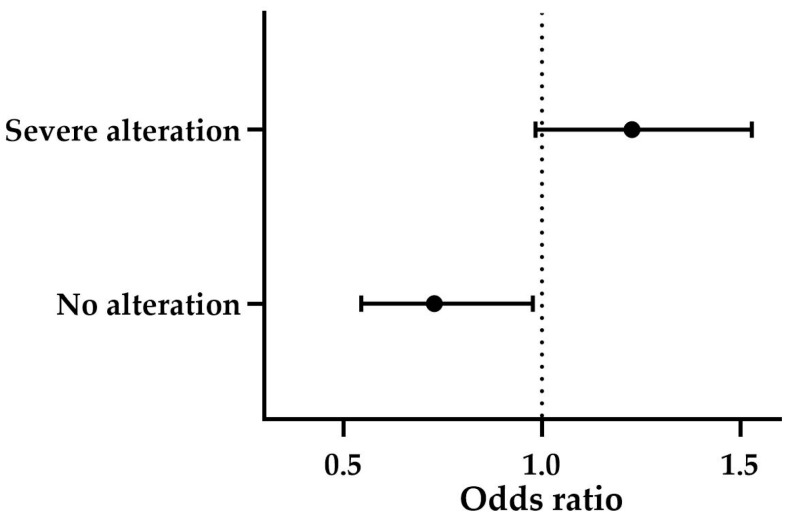
Forest plot showing the odds ratios (ORs) and 95% confidence intervals (CIs) for the predictive value of CPSE levels on the degree of ultrasonographic structural alteration. The solid circles represent the ORs, and the whiskers indicate the CIs. The dotted line at OR = 1 serves as a reference. The OR greater than 1 indicates a positive predictive effect, while the lower than one a negative prediction. CPSE = canine prostatic specific esterase.

Finally, the overall categorical assessment revealed a significant association between the groups and the degree of structural alteration (*p* ≤ 0.001). Subclinical BPH was predominantly associated with mild alterations (71%), while clinical BPH and BPH-Prostatitis both exhibited a high prevalence of severe alterations, with 61.5% and 77.8%, respectively. According to the statistically significant multinomial logistic regression model (*p* ≤ 0.001), higher CPSE levels showed a trend toward predicting severe alterations (OR = 1.227, B = 0.204, *p* = 0.069). Similarly, when high CPSE levels were present, the likelihood of not detecting structural changes was low (OR = 0.729, B = −0.316, *p* ≤ 0.05) (Figure 7). The effect of age was not significant (*p* > 0.05).

## 4. Discussion

Previous publications stated that the evolution of BPH is individual and difficult to predict [6]. The findings of this study highlight the potential of CPSE as a marker for BPH progression, integrating multiple key characteristics of the disorder rather than relying solely on clinical signs, as validated clinical indexes do [26], or on ultrasonographic findings, as previous approaches have suggested [5,29]. According to the results of our predictive models for clinical severity, complexity, and structural alteration, CPSE can provide a comprehensive and integrative assessment of BPH progression, ultimately supporting more precise and personalized clinical decision-making.

Most dog-based studies have focused on evaluating the utility of CPSE for diagnosing BPH, in general, or for detecting its subclinical stages [4,15,16,30]. However, this straightforward application of CPSE may not be the most reliable, given the range of threshold values proposed across the literature [4,16]. When interpreting our CPSE findings in the context of these thresholds, we observed that the median level for subclinical BPH (56.33 ng/mL) aligns with values such as the 52.3 ng/mL reported by Alonge et al. (2018) [4] yet contrasts with higher thresholds like the 82.56 ng/mL suggested by Laurusevičius et al. (2024) [16]. Additionally, our study showed no significant difference in CPSE levels between control dogs and those with subclinical BPH, further supporting the inconsistency of CPSE values reported for this disease stage. Even among symptomatic dogs, the median CPSE levels (clinical BPH: 74.67 ng/mL, IQR: 62.44–96.33; BPH-prostatitis: 81.89 ng/mL, IQR: 65.78–116.89) were close to, but not consistently above, the 90 ng/mL threshold commonly cited for clinically manifesting cases [30]. This ongoing variability supports our approach of using CPSE as an indicator of clinical or structural changes over time, rather than as a definitive tool for stage differentiation.

In fact, clinical decision-making in BPH cases is often less focused on classifying the disease and more oriented toward anticipating its potential consequences. Currently, the only validated methods to assess disease progression during BPH involve clinical scoring systems like the ZIS, which was adapted by Ruetten et al. (2021) [8] in one of their studies. In line with our findings, their study did not report any cases scoring above 34—the threshold for severe symptomatology [8]. The median ZIS for symptomatic BPH in that study was 14.5, closely aligning with the values observed in our clinical BPH and BPH-Prostatitis groups. Mild clinical severity was predominant in both studies, indicating a generalizable trend. Encouragingly, our data also showed a significant correlation between CPSE levels and ZIS, supporting the utility of CPSE as a reliable marker for evaluating clinical progression.

In line with the work of Polisca et al. (2016) [31] and Cazzuli et al. (2023) [18], who provided valuable insights into the clinical complexity of prostatic pathologies, our study extends this understanding by exploring the association between CPSE levels and specific symptom profiles, as well as by proposing a predictive model for disease progression. Our findings reveal that elevated CPSE levels correlate with both an increased number of symptoms and greater heterogeneity among symptom categories, suggesting that rising CPSE may signal a transition in clinical presentation, such as the emergence of urinary symptoms in patients initially presenting with digestive signs, or vice versa, aligning with the dynamic relationships between CPSE and clinical evolution discussed by Pinheiro et al. (2017) [15].

According to the results presented in this study, CPSE appears to be a relevant predictor of symptom severity and complexity in BPH and, therefore, of clinical progression. The relevance of these findings lies in how BPH is assessed in practical settings. Same clinical signs may persist for extended periods, and treatment is not always initiated unless these signs significantly affect the patient’s quality of life [32]. In this context, and consistent with strategies employed in human medicine, some patients are managed under a “watchful waiting” approach, with routine monitoring every 3 to 6 months and no immediate therapeutic intervention [32,33]. A major limitation of this strategy is the inability to reliably anticipate whether the disease will remain stable or progress in the short term. The predictive value of CPSE in relation to clinical severity and complexity offers a potential solution to this gap. Elevated CPSE levels in conservatively managed patients may serve as early indicators of imminent disease progression, thereby supporting more proactive clinical decision-making. In such cases, clinicians may opt for increased monitoring frequency or earlier treatment initiation, even when symptoms are mild. Furthermore, a higher likelihood of a faster progression toward more severe or complex forms may influence treatment choice. Osaterone acetate is associated with more rapid symptom relief, whereas finasteride requires a longer treatment duration of approximately 30 days to achieve comparable outcomes [34]. In patients with elevated CPSE levels, indicating a likely short-term worsening of clinical status, a faster-acting treatment may be warranted. However, a limitation of the present study is the absence of a clearly defined CPSE threshold to guide such decisions. Further research is needed to establish clinically meaningful cut-off values.

The association between elevated CPSE levels and increased prostate volume, as demonstrated in our study, has also been reported by other investigations [4,15,30]. However, the degree of volume increase associated with specific CPSE levels or the clinical/subclinical type of BPH varies across studies. For instance, Holst et al. (2017) identified a prostate volume ratio exceeding 2.5 (and CPSE > 90 ng/mL) as indicative of clinically manifested BPH, while Alonge et al. (2018) observed a 1.5-fold increase in subclinical BPH cases (along a CPSE level > 50 ng/mL) [4,30]. In contrast, our study found that actual/expected volume ratios slightly above 1 were present not only in subclinical BPH cases but also in clinically manifesting forms of BPH. However, this discrepancy may be explained by differences in measurement methodologies, such as the use of varying formulas for volume calculation.

The dynamics of ultrasonographic prostatic structure in BPH have previously been investigated using similar evaluation criteria as in our study, particularly in the context of monitoring treatment-induced changes [19]. Building on these previous studies, our results suggest that CPSE levels may serve as a potential marker for predicting structural alterations in BPH cases. In relation to the findings by Niżański et al. (2020) [19], our study indicates that CPSE may also have potential as a tool for monitoring therapeutic response. However, further studies are required to confirm the statistical significance of this trend.

Further detailed analysis of specific ultrasonographic features strengthened the relationship between CPSE levels and prostatic ultrasound structure. To enhance the objectivity of the structural assessment, we employed pixel-based image analysis of parenchymal stippling patterns, moving beyond basic categorizations like “regular”, “coarse”, or “increased”. Stippled areas became more pronounced as the condition progressed from control dogs to subclinical and, subsequently, to clinical BPH cases. This trend was mirrored by increasing CPSE levels, reinforcing the association between echotextural changes, BPH progression, and CPSE. The relevance of this finding is further supported by strong positive correlations between the extent of stippled areas and clinical complexity, severity, and ZIS.

Although previous studies have explored the presence of cysts and other ultrasonographic alterations in relation to CPSE levels [4,35], the relationship between CPSE concentration and cyst size has received less attention. The existing literature has focused on the total number of the cysts or the general presence of these structures rather than their dimensions [4,15,16,30]. This is an important gap, as the size of prostatic cysts can influence therapeutic outcomes [34]. For instance, cysts smaller than 0.5 cm tend to resolve after 60 days of finasteride treatment [36], while osaterone acetate has been shown to be effective in cases where cysts do not exceed 1 cm in diameter [19]. Our study contributes to this area by demonstrating a positive correlation between CPSE levels and the size of intraprostatic cysts/abscesses, suggesting that CPSE may serve as a useful marker in guiding treatment decisions. This is the first study to investigate CPSE levels in relation to the presence of paraprostatic cysts/abscesses. We found no significant correlation between these structures and CPSE levels, which is consistent with their distinct pathogenesis. Unlike intraprostatic lesions, paraprostatic cysts/abscesses are believed to arise from remnants of the cystic uterus masculinus [37,38] and, therefore, may not reflect the same biological processes influencing CPSE production.

Age is typically considered a strong confounder in BPH diagnosis in dogs [5], as it has been previously shown to be correlated with BPH progression. This explains the positive correlation between age and CPSE levels observed in our population. However, despite this correlation, age did not significantly influence the predictive models for clinical complexity, severity, or structural alterations. While age may be related to a higher likelihood of developing BPH, apparently it cannot anticipate how the disorder will progress.

Certain breeds are known to be predisposed to BPH [31]. However, due to the diverse breed composition of our study population, it was not feasible to include breed as a confounder in our models, even when categorized. This is a limitation of the study, as a more homogenous population in terms of breed composition would have been more suitable for prediction models analysis.

Another potential limitation of the study is the possibility of inadvertently including cases of prostatic carcinoma within the groups. Prostatic carcinoma typically presents with distinct ultrasonographic features, including loss of parenchymal architecture, disruption of the capsule, and mineralization foci [9]. Group assignment for cases with ultrasonographic changes was based on cytology, which shows approximately 80% agreement with histopathology for the diagnosis of prostatic carcinoma [39]. Although this level of agreement was considered acceptable for our study design, the absence of histopathological confirmation requires us to acknowledge this as a limitation.

## 5. Conclusions

In summary, CPSE proves to be a reliable predictor for assessing disease progression in addition to serving as a biomarker for BPH diagnosis in dogs. The study demonstrates significant associations between CPSE levels and clinical severity, complexity, and prostatic ultrasound alterations. These findings underscore CPSE’s potential to provide a comprehensive evaluation of BPH beyond conventional diagnostic tools, supporting more informed clinical decision-making. However, based on our results, some limitations exist regarding CPSE’s ability to clearly differentiate between healthy and subclinical dogs, as well as between clinical BPH and BPH-Prostatitis cases, due to overlapping values. Nonetheless, CPSE offers valuable insights into the disease’s trajectory, highlighting its role in guiding therapeutic interventions and enhancing disease monitoring in veterinary practice.

## Data Availability

All data presented in this study are available upon request from the corresponding author.

## Supplementary Materials

The following supporting information can be downloaded at: https://www.mdpi.com/article/10.3390/ani15111614/s1, Table S1: Prostate-related clinical symptoms assessed and grouped according to Cazzuli et al. (2023) [18]; Table S2: Age, breed and weight distribution among all included dogs.

## Abbreviations

The following abbreviations are used in this manuscript:

BPH	Benign prostatic hyperplasia
CPSE	Canine prostate-specific esterase
PSA	Prostate-specific antigen
L	Length
W	Width
DL	Depth on the longitudinal scan
DT	Depth on the transverse scan
A	Age
BW	Body weight
FNA	Fine needle aspiration
ZIS	Zambelli index score
ROI	Region of interest
ELISA	Enzyme-linked immunosorbent assay
IQR	Interquartile range
OR	Odds ratio
CI	Confidence interval
