# Supplementary material for: Is Canine Prostate-Specific Esterase a Reliable Marker for Benign Prostatic Hyperplasia Progression in Dogs?
Source: Animals (Basel). 2025 May 30;15(11):1614. doi: 10.3390/ani15111614 (PMC12153740; doi:10.3390/ani15111614)
Supplement: Supplementary file 1 [file animals-15-01614-s001.zip › animals-3651961-supplementary.pdf]

Table S1. Prostate-related clinical symptoms assessed and grouped according to Cazzuli et al. (2023).

**Symptom category**			
Digestive signs	Tenesmus<br>Anorexia<br>Weight loss	Constipation<br>Hematochezia<br>Vomiting	Diarrhea<br>Dyschezia<br>Flat stools
Urinary signs	Polyuria/polydipsia<br>Hematuria<br>Dysuria	Blood dripping from the urethra<br>Urinary incontinence<br>Stranguria	Anuria<br>Pollakiuria
Others signs	Lethargy<br>Perineal hernia<br>Hematospermia	Abdominal pain from palpation<br>Hind limb weakness<br>Difficulty with locomotion	Prostatomegaly / pain during rectal examination

Table S2. Age, breed and weight distribution among all included dogs.

Group	Breed	Age (months – years)	Weight (kg)
Control	Australian Shepherd	14 - 1.17	26.7
	Beauceron	89 - 7.42	21.4
	Belgian Malinois	39 - 3.25	33.9
	Border Collie	72 - 6.00	18.3
	Border Collie	45 - 3.75	16.2
	Border Terrier	24 - 2.00	9.0
	English Springer Spaniel	42 - 3.50	21.7
	Flatcoated Retriever	84 - 7.00	40.2
	German Shepherd	58 - 4.83	46.3
	German Shepherd	37 - 3.08	30.1
	Golden Retriever	16 - 1.33	34.0
	Golden Retriever	84 - 7.00	39.2
	Labradoodle	90 - 7.50	23.2
	Labrador Retriever	16 - 1.33	38.0
	Labrador Retriever	13 - 1.08	29.5
	Labrador Retriever	15 - 1.25	36.5
	Labrador Retriever	18 - 1.50	28.8
	Miniature Australian Shepherd	15 - 1.25	8.9
	Rough Collie	24 - 2.00	21.0
	Shetland Sheepdog	85 - 7.08	8.7
	Shetland Sheepdog	32 - 2.67	6.8
	White Swiss Shepherd	70 - 5.83	36.4
Subclinical BPH	Beagle	37-3.08	14.8
	Border Collie	112-9.33	12.1
	Border Terrier	108-9.00	10.4
	Braque Français	58-4.83	24.0
	Cavalier King Charles Spaniel	62-5.17	7.5
	Dachshund	49-4.08	9.2
	Drentse Patrijshond	39-3.25	30.4
	German Shepherd	40-3.33	34.5
	Irish Setter	72-6.00	26.3
	Irish Setter	114-9.50	27.7
	Mixed breed	52-4.33	25.3
	Rhodesian Ridgeback	52-4.33	40.6
	Scottish Terrier	24-2.00	8.7
	Staffordshire Bull Terrier	84-7.00	27.1
Clinical BPH	American Staffordshire Terrier	156-13.00	14.6
	American Staffordshire Terrier	68-5.67	28.2
	American Staffordshire Terrier	38-3.17	28.0
	American Staffordshire Terrier	54-4.50	29.0
	Beauceron	136-11.33	31.3
	Beauceron	89-7.42	43.1
	Boxer	126-10.50	35.0
	Chinese Crested Dog	55-4.58	4.2
	Cocker Spaniel	158-13.17	15.5
	Flatcoated Retriever	108-9.00	43.0
	German Pointer	65-5.42	21.1
	Labradoodle	36-3.00	15.2

	Mixed breed	35-2.92	34.4
	Mixed breed	138-11.50	5.2
	Mixed breed	146-12.17	9.5
	Mixed breed	121-10.08	12.3
	Mixed breed	86-7.17	17.4
	Mixed breed	144-12.00	15.3
	Nova Scotia Duck Tolling Retriever	113-9.42	17.3
	Rottweiler	73-6.08	58.0
	Rottweiler	120-10.00	51.5
	Shih Tzu	108-9.00	5.9
	Siberian Husky	105-8.75	33.5
	Small Munsterlander	61-5.08	25.3
	Vikhan Sheepdog	102-8.50	46.7
	White Swiss Shepherd	154-12.83	38.7
BPH-Prostatitis	American Staffordshire Terrier	68-5.67	28.9
	Boxer	132-11.00	29.7
	Boxer	66-5.50	35.0
	Cane Corso	60-5.00	51.3
	English Bulldog	100-8.33	21.0
	Mixed breed	140-11.67	14.5
	Mixed breed	151-12.58	49.2
	Siberian Husky	123-10.25	32.1
	Transylvanian Hound	144-12.00	29.6

BPH - benign prostatic hyperplasia
